# Accumulated Expression Level of Cytosolic *Glutamine Synthetase 1* Gene (*OsGS1;1* or *OsGS1;2*) Alter Plant Development and the Carbon-Nitrogen Metabolic Status in Rice

**DOI:** 10.1371/journal.pone.0095581

**Published:** 2014-04-17

**Authors:** Aili Bao, Zhuqing Zhao, Guangda Ding, Lei Shi, Fangsen Xu, Hongmei Cai

**Affiliations:** 1 Microelement Research Center, Huazhong Agricultural University, Wuhan, China; 2 Key Laboratory of Arable Land Conservation (Middle and Lower Reaches of Yangtse River), Ministry of Agriculture, Huazhong Agricultural University, Wuhan, China; 3 National Key Laboratory of Crop Genetic Improvement, Huazhong Agricultural University, Wuhan, China; University of Nebraska-Lincoln, United States of America

## Abstract

Maintaining an appropriate balance of carbon to nitrogen metabolism is essential for rice growth and yield. Glutamine synthetase is a key enzyme for ammonium assimilation. In this study, we systematically analyzed the growth phenotype, carbon-nitrogen metabolic status and gene expression profiles in *GS1;1*-, *GS1;2*-overexpressing rice and wildtype plants. Our results revealed that the *GS1;1*-, *GS1;2*-overexpressing plants exhibited a poor plant growth phenotype and yield and decreased carbon/nitrogen ratio in the stem caused by the accumulation of nitrogen in the stem. In addition, the leaf SPAD value and photosynthetic parameters, soluble proteins and carbohydrates varied greatly in the *GS1;1*-, *GS1;2*-overexpressing plants. Furthermore, metabolite profile and gene expression analysis demonstrated significant changes in individual sugars, organic acids and free amino acids, and gene expression patterns in *GS1;1*-, *GS1;2*-overexpressing plants, which also indicated the distinct roles that these two *GS1* genes played in rice nitrogen metabolism, particularly when sufficient nitrogen was applied in the environment. Thus, the unbalanced carbon-nitrogen metabolic status and poor ability of nitrogen transportation from stem to leaf in *GS1;1-*, *GS1;2*-overexpressing plants may explain the poor growth and yield.

## Introduction

Nitrogen is an essential macronutrient required for plant growth and development, and it is a major limiting factor in determining plant productivity and crop yield [1]–[4]. Carbon is crucial for plants to perform their routine and fundamental cellular activities. Carbon compounds include various carbohydrates; in particular, sucrose, glucose and organic acids provide both the energy and the carbon skeletons for ammonium (NH_4_^+^) assimilation during amino acid biosynthesis. Amino acids and the resulting proteins, in particular enzymes, are essential for nearly all cellular activities [5]. In addition to their independent utilization, the coordination and optimal functioning of the metabolic pathways for nitrogen and carbon assimilation in plants are critical for determining plant growth and, ultimately, biomass accumulation [6], [7]. In addition, maintaining an appropriate balance or ratio of carbohydrates to nitrogen metabolites in the cell, which is referred to as the “carbon/nitrogen balance,” is also important for the regulation of plant growth, development and yield production [5], [8]–[10].

In higher plants, glutamine synthetase (GS; EC 6.3.1.2) is a key enzyme for the assimilation of ammonium into glutamine (Gln) [11]–[13]. The reaction of GS is coupled with glutamate synthase (GOGAT), which generates two molecules of glutamate (Glu). One molecule of glutamate is cycled back to the GS reaction as a substrate, and the other molecule of glutamate is exported or used to produce other amino acids [14]. Two major isoforms of GS, GS1 in the cytosol and GS2 in plastids, are present in higher plants [15], [16]. A multigene family encodes the cytosolic isoform GS1, and a single gene encodes the plastidic isoform GS2, although several *GS2* genes have been identified in soybean, alfalfa and durum wheat [17]–[23]. In rice, there are three genes that encode cytosolic GS1 (*OsGS1;1*, *OsGS1;2* and *OsGS1;3*), and one gene encodes chloroplastic GS2 (*OsGS2*) [2]. *OsGS1;1* is mainly expressed in the shoot, *OsGS1;2* mRNA is abundant in the root, *OsGS1;3* mRNA is present only in the spikelets, and *OsGS2* is mainly expressed in the leaf [24].

Numerous studies of the GS enzyme have emphasized the importance of this enzyme in plant nitrogen metabolism. Previous reports have shown that overexpression of GS genes aimed to improve plant nitrogen assimilation presented variable results. For example, accelerated growth rate was observed in transgenic *Lotus corniculatus* plants overexpressing a soybean GS1 isoenzyme driven by the *CaMV* 35S promoter [25]. In addition, vegetative growth and photosynthetic capacity improvements were reported for cytosolic *GS1*-overexpressed poplar trees [26]–[29], tobacco [30]–[32] and *L. japonicus* [33]. Earlier flower and seed development were also observed in transgenic wheat lines containing *Phaseolus vulgaris GS1* under the control of the *Rubisco* small subunit (*rbcS*) promoter [34]. In addition, a high level of GS activity in the root was negatively correlated with aboveground biomass in *Lotus japonicus* [35]. However, pea plants overexpressing a soybean *GS1* under the control of a root-specific promoter showed no consistent effect on total biomass [36]. Furthermore, recent studies of mutants deficient in leaf cytosolic GS in rice, maize and *Arabidopsis* demonstrated the important role of cytosolic GS in nitrogen remobilization for grain filling, carbon and nitrogen metabolic coordination, and ammonium homeostasis. In rice, homozygous lines of three *OsGS1;1*-knockout mutants showed a decrease in cytosolic GS enzyme activity, delay in growth rate, reduced spikelet weight and number, and reduced fertility [24]. Metabolic profile analyses revealed an imbalance in the levels of sugars, amino acids and metabolites in the tricarboxylic acid cycle and an over-accumulation of secondary metabolites in *OsGS1;1*-knockout mutants [37]. In maize, *gln1–4* mutants exhibited a reduced kernel size and the *gln1–3* mutant exhibited a reduced kernel number, a higher free amino acid content and a lower carbon/nitrogen ratio with no effect on the shoot dry weight, even in double mutants [38], [39]. In *Arabidopsis*, *gln1;2* knockout mutants displayed lower glutamine synthetase activity, higher ammonium concentration, and reduced rosette biomass compared with the wildtype (WT) under conditions of ample nitrate supply [40].

Because rice growth and yield requires abundant nitrogen, large amounts of nitrogen fertilizers are used. However, crop plants use less than half of the nitrogen fertilizers applied [41]. The unused nitrogen is inevitably leached into the underground water system and lost to the atmosphere, resulting in severe environmental pollution. Recent analyses have demonstrated that soil acidification in China primarily resulted from high-N fertilizer inputs [42]. To improve nitrogen use efficiency, the rice yield and/or to ensure normal plant growth and yield under low nitrogen fertilizer supplies, we generated *OsGSs*-overexpressing transformants driven by the *CaMV*35S promoter and obtained transgenic rice plants using the *Agrobacterium-*mediated transformation method in the previous study [43], [44]. The *GS1;1*-, *GS1;2*-overexpressing plants displayed an imperceptible growth phenotype at the seedling stage when grown hydroponically under both normal and low nitrogen conditions and showed decreases in both grain yield and total amino acids in seeds grown in fields with low nitrogen fertilizer [43]. To identify the reasons for these observations, we systematically analyzed differences in the growth, carbon-nitrogen metabolic status and gene expression profiles between the *GS1;1*-, *GS1;2*-overexpressing rice and wildtype Zhonghua 11 at different developmental stages grown under different nitrogen levels. The results in this study revealed that overexpressing *GS1;1*, *GS1;2* genes altered plant growth and development, yield, carbon and nitrogen metabolism. The imbalance in carbon and nitrogen may be the main reason for the decreased yield in *GS1;1*-, *GS1;2*-overexpressing plants.

## Materials and Methods

### Plant Materials and Growth Conditions

Two cDNA sequences encoding the rice cytosolic *GS1;1* (AB037595) and *GS1;2* (AB180688) were isolated from the Minghui 63 normalized cDNA library (http://www.redb.ncpgr.cn). *GS* fragments were then ligated into the *pCAMBIA* 1301 S vector, driven by cauliflower mosaic virus (*CaMV*) 35 S promoter. The chimeric gene was transformed into the *japonica* rice cultivar Zhonghua 11 by an *Agrobacterium tumefaciens*-mediated transformation method. The *GS* expression level and copy numbers of the transgene were analyzed by the Northern blot and Southern blot techniques in the T_0_ generation which were described in the previous study [43].

Seeds of *GS1;1*, *GS1;2*-overexpressing rice in the T_3_ generation and wildtype Zhonghua 11 (*Oryza sativa* ssp. *japonica*) were germinated and sown in sand. At the 2-leaf stage, the seedlings were transferred into a normal nutrient solution containing 1.44 mM NH_4_NO_3_, 0.3 mM NaH_2_PO_4_, 0.5 mM K_2_SO_4_, 1.0 mM CaCl_2_, 1.6 mM MgSO_4_, 0.17 mM Na_2_SiO_3_, 50 µM Fe-EDTA, 0.06 µM (NH_4_)_6_Mo_7_O_24_, 15 µM H_3_BO_3_, 8 µM MnCl_2_, 0.12 µM CuSO_4_, 0.12 µM ZnSO_4_, 29 µM FeCl_3_ and 40.5 µM citric acid, pH 5.5 [45]. The culture solution was refreshed every 3 days. After a week, the plants were transferred into a culture solution without NH_4_NO_3_ (0×N treatment), a culture solution with 1/10 NH_4_NO_3_ (0.1×N treatment), a culture solution with 5-fold NH_4_NO_3_ (5×N treatment), and a culture solution with complete nutrients (1×N treatment). The plant materials were harvested at the tillering stage and the heading stage for analysis of the growth phenotype, leaf SPAD value, photosynthesis, carbon and nitrogen content, concentration of soluble proteins and carbohydrates, metabolic profiling and gene expression. Moreover, seedlings of the *GS1;1*, *GS1;2*-overexpressing rice and wildtype Zhonghua 11 were transferred into pots fertilized with 0.15 g P_2_O_5_/kg soil and 0.2 g K/kg soil (0×N treatment), pots fertilized with 0.02 g N/kg soil, 0.15 g P_2_O_5_/kg soil and 0.2 g K/kg soil (0.1×N treatment), pots fertilized with 0.2 g N/kg soil, 0.15 g P_2_O_5_/kg soil and 0.2 g K/kg soil (1×N treatment), and pots fertilized with 1 g N/kg soil, 0.15 g P_2_O_5_/kg soil and 0.2 g K/kg soil (5×N treatment). At the mature stage, the yield production was analyzed. All the plants were grown in the pot farm in Huazhong Agricultural University, Wuhan, China.

### Nitrogen Uptake Assay

Seeds of the *GS1;1*, *GS1;2*-overexpressing rice and wildtype Zhonghua 11 (*Oryza sativa* ssp. *japonica*) germinated and were sown in sand. At the 2-leaf stage, the seedlings were transferred into the normal nutrient solution as previously described [45]. At the tillering stage, the NH_4_NO_3_ in the nutrient solution was replaced with ^15^NH_4_^15^NO_3_. Six samples of randomly mixed plant root, stem and leaf materials were harvested after 1 h, 3 h, 8 h, 1 d and 3 d. The ^15^N content was analyzed using an isotope mass spectrometer (ANCA-MS, Europa Scientific, Crewe, UK) according to the manufacturer’s instructions. The total carbon and nitrogen content in the root, stem and leaves of *GS1;1*-, *GS1;2*-overexpressing plants and wildtype plants under the 0×N, 0.1×N, 1×N and 5×N conditions at the tillering stage was determined using a C/N analyzer (Elementar, Vario MAX CN, Germany) according to the manufacturer’s instructions, with L-glutamic acid as a standard.

### Determination of the Leaf SPAD Value and Photosynthetic Parameters

At both the tillering stage and heading stage, every ten plants of the *GS1;1*, *GS1;2*-overexpressing rice and wildtype Zhonghua 11 grown under the 0×N, 0.1×N, 1×N and 5×N conditions were randomly selected for the determination of the leaf SPAD value. A chlorophyll meter (SPAD-502) was used to test the SPAD value of the upper, middle and bottom portion of the flag leaf of each plant according to the manufacturer’s instructions, and the average mean was used in the data analysis. At the heading stage, every ten plants of the *GS1;1*, *GS1;2*-overexpressing rice and wildtype Zhonghua 11 grown under the 0×N, 0.1×N, 1×N and 5×N conditions were randomly selected for the determination of the photosynthetic parameters. A Li-6400XT portable photosynthesis system (USA, Li-COR) was used to test the photosynthetic rate, stomatal conductance, intercellular CO_2_ concentration and transpiration rate of the upper, middle and bottom portion of the flag leaf of each plant according to the manufacturer’s instructions, and the average mean was used in the data analysis.

### Determination of the Physiological Parameters

For the soluble protein and carbohydrate analysis, three samples of randomly mixed plant root, stem and leaf materials from three biological replications were harvested at the tillering stage and heading stage. The plant materials were homogenized by grinding the freshly harvested leaves on ice with extraction buffer [10 mM Trizma (pH 7.5), 5 mM sodium glutamate, 10 mM MgSO_4_, 1 mM dithiothreitol, 10% (v/v) glycerol, and 0.05% (v/v) Triton X-100]. The homogenates were then centrifuged at 12,000 *g* for 20 min at 4°C [46]. The soluble protein concentration of the extract was measured using the Bradford [47] protein assay and Coomassie Plus Protein Assay Reagent (Pierce, Rockford, IL, USA); bovine serum albumin was used as the standard protein. The soluble carbohydrates were extracted from pre-dried plant materials with boiling water and colorimetrically measured according to the anthrone procedure [48], [49]. For the metabolite profiling analysis, samples of six randomly mixed plant root and leaf materials of the *GS1;1*, *GS1;2*-overexpressing rice and wildtype Zhonghua 11 were harvested at the tillering stage and analyzed using the GC-TOF-MS method. Extracts from 200 mg FW samples were used. The data pre-treatment and normalization, alignments and metabolite identification were performed as previously described by Kusano et al. [50] and Redestig et al. [51].

### Gene Expression Analysis

For gene expression analysis, both root and leaf materials of the *GS1;1*, *GS1;2*-overexpressing rice and wildtype Zhonghua 11 were harvested from three biological replications under 0×N, 0.1×N, 1×N and 5×N conditions at the tillering stage. Total RNA was extracted with TriZol reagent (Invitrogen, Germany) according to the manufacturer's instructions. For q-RT PCR analysis, first-strand cDNAs were synthesized from DNaseI-treated total RNA using Superscript II reverse transcriptase (Invitrogen) according to the manufacturer’s instructions. Q-RT PCR was performed in an optical 96-well plate with an ABI PRISM 7500 real-time PCR system (Applied Biosystems, Foster City, CA, USA). Each reaction contained 12.5 µl of 2×SYBR Green Master Mix reagent (Applied Biosystems), 3.0 µl of cDNA, and 200 µM each of the gene-specific primers in a final volume of 25 µl. The thermal cycle used was as follows: 95°C for 3 min followed by 45 cycles of 95°C for 30 s, 60°C for 30 s, and 72°C for 40 s. All gene-specific primers for q-RT PCR are designed on the basis of the cDNA sequences and listed in Table S1. The specific primer for the rice *actin* gene (NM_197297) was used as an internal control. The primers were designed using Primer Express Software (Foster City, CA, USA) and checked using the BLAST program with the rice genomic sequence available in the database of the Institute for Genomic Research (TIGR, http://rice.plantbiology.msu.edu/) to ensure that the primers would amplify a unique and desired cDNA segment. The specificity of the reactions was checked by melting curve analysis, and three replicates of each cDNA sample were used for q-RT PCR analysis.

## Results

### Growth Phenotype and Yield Analysis in GS1-overexpressing Plants

In our previous study, we obtained *GS1;1-* and *GS1;2*-overexpressing rice plants using the *Agrobacterium*-mediated transformation method, and we failed to observe obvious differences in the growth phenotype between transgenic plants and wildtype plants at the seedling stage when grown hydroponically under normal and low nitrogen conditions [43]. To determine whether the accumulated mRNA transcript level of *GS1;1* or *GS1;2* will affect the development and productivity in transgenic plants, we analyzed the root length, plant height, root and shoot dry weight, leaf SPAD value and photosynthetic parameters of *GS1;1-*, *GS1;2*-overexpressing plants and wildtype plants at the tillering stage and heading stage grown hydroponically under four different nitrogen levels (0×N, 0.1×N, 1×N and 5×N). The yields of *GS1;1-*, *GS1;2*-overexpressing plants and wildtype plants at the mature stage growth in pots were also tested.

**Figure 1:**
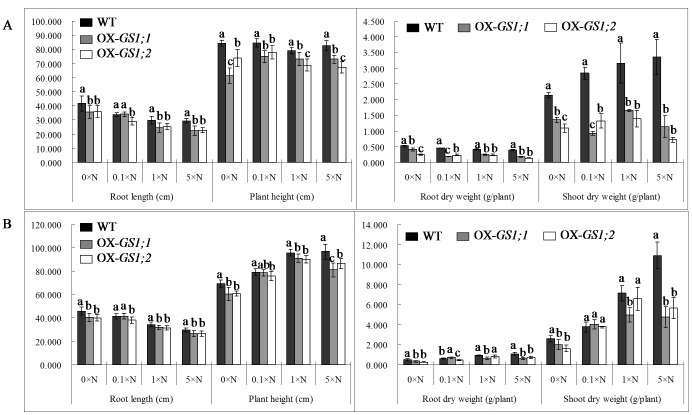
The root length, plant height, root and shoot dry weight in the *GS1;1*-, *GS1;2*-overexpressing plants (OX-*GS1;1*, OX-*GS1;2*) and wildtype plants (WT) at the tillering stage (A) and the heading stage (B) under 0×N, 0.1×N, 1×N and 5×N conditions. Values are the mean ± SD of ten randomly selected plants. a, b, c indicate the significant difference at the level of P = 0.05.

Our results showed a significant (P<0.05) decrease in the root length and plant height, root and shoot dry weight in *GS1;1-*, *GS1;2*-overexpressing plants when compared to wildtype plants at both the tillering and heading stages under the 0×N, 0.1×N, 1×N and 5×N conditions (Fig. 1). Compared to wildtype plants, at the tillering stage, *GS1;1*-overexpressing plants demonstrated 14.4–23.9%, 7.4–27.2%, 19.8–58.2% and 36.7–67.5% decreases in the root length, plant height, root and shoot dry weight, respectively; for *GS1;2*-overexpressing plants, there were 13.5–22.8%, 7.9–18.7%, 43.6–64.9% and 48.8–78.6% decreases in the root length, plant height, root and shoot dry weight, respectively (Fig. 1A). At the heading stage, for *GS1;1*-overexpressing plants, there were 7.2–11.7%, 0.7–16.0%, 30.2–44.0% and 21.7–56.1% decreases in the root length, plant height, root and shoot dry weight, respectively; for *GS1;2*-overexpressing plants, there were 7.5–12.5%, 4.1–12.1%, 13.1–42.8% and 0.1–78.5% decreases in the root length, plant height, root and shoot dry weight, respectively (Fig. 1B).

During the leaf SPAD value determination, compared to wildtype plants, there were significant (P<0.05) decreases in *GS1;1*-overexpressing plants at the heading stage under the 1×N (4.5% decrease) and 5×N (5.8% decrease) conditions, while there were significant (P<0.05) increases in *GS1;2*-overexpressing plants at the tillering stage under the 0×N (7.0% increase) and 0.1×N (5.7% increase) conditions (Table 1). During photosynthesis analysis at the heading stage, there were significant (P<0.05) changes in *GS1;1*-overexpressing plants, while no significant (P<0.05) changes in *GS1;2*-overexpressing plants were observed when compared to wildtype plants. For example, there was a 12.4% decrease in intercellular CO_2_ concentration and 21.5% decrease in transpiration rate under the 0.1×N condition, a 36.6% increase in stomatal conductance and 18.0% increase in transpiration rate under the 1×N condition, and 34.0% increase in stomatal conductance and 32.6% increase in transpiration rate under the 5×N condition in *GS1;1*-overexpressing plants compared to wildtype plants at the heading stage (Table 1). However, no significant (P<0.05) changes in photosynthetic rate were observed in *GS1;2* transgenic plants (Table 1).

**Table 1:** The leaf SPAD value and photosynthetic parameters in the *GS1;1*-, *GS1;2*-overexpressing plants (OX-*GS1;1*, OX-*GS1;2*) and wildtype plants (WT) at the tillering stage and the heading stage under 0×N, 0.1×N, 1×N, 5×N conditions.

	SPAD		Photosynthetic parameters at heading stage			
	Tilleringstage	Headingstage	Photosynthetic rate(µmol CO_2_ m^−2^ s^−2^)	Stomatal conductance(mmol m^−2^ s^−1^)	Intercellular CO_2_concentration (µl L^−1^)	Transpiration rate(mmol H_2_O m^−2^ S^−1^)
**0×N**						
WT	34.3±2.4 b	32.7±1.7 a	13.24±1.47 a	0.28±0.04 a	280.48±1.12 a	7.54±0.65 a
OX-*GS1;1*	34.0±1.9 b	32.4±2.0 a	14.05±2.34 a	0.30±0.04 a	281.09±12.52 a	8.06±0.91 a
OX-*GS1;2*	36.7±1.9 a	33.6±1.2 a	12.56±1.68 a	0.27±0.03 a	282.18±3.46 a	7.59±0.84 a
**0.1×N**						
WT	40.7±2.0 b	40.7±2.2 a	10.58±1.64 a	0.29±0.02 a	297.52±10.16 a	7.24±0.25 a
OX-*GS1;1*	41.5±1.2 ab	40.1±1.4 a	12.13±1.44 a	0.25±0.06 a	260.48±9.12 b	5.68±0.79 b
OX-*GS1;2*	43.0±1.8 a	41.6±1.6 a	11.28±2.82 a	0.28±0.02 a	296.19±14.99 a	6.65±0.66 a
**1×N**						
WT	43.6±2.0 a	47.1±0.5 a	16.07±1.78 a	0.41±0.04 b	290.97±8.26 a	7.49±0.47 b
OX-*GS1;1*	44.1±3.1 a	45.0±0.9 b	18.23±2.44 a	0.56±0.08 a	300.02±12.95 a	8.84±0.81 a
OX-*GS1;2*	44.1±1.8 a	46.2±0.6 a	13.87±0.50 a	0.35±0.03 b	294.62±8.63 a	7.69±0.20 b
**5×N**						
WT	44.4±1.5 a	48.4±1.3 a	19.12±3.45 a	0.53±0.05 b	305.62±15.74 a	7.85±0.17 b
OX-*GS1;1*	44.2±2.1 a	45.6±2.1 b	21.57±2.87 a	0.71±0.07 a	302.61±11.59 a	10.41±0.61 a
OX-*GS1;2*	43.3±3.0 a	45.4±2.7 b	17.01±3.48 a	0.44±0.12 b	293.89±2.79 a	7.81±1.11 b

Values are mean ± SD from ten randomly selected plants. a, b indicate the significant difference at the level of P = 0.05.

During the yield analysis at the mature stage, compared to wildtype plants, significant (P<0.05) decreases in both *GS1;1-* and *GS1;2*-overexpressing plants were observed, except the yield in *GS1;2*-overexpressing plants under 0.1×N condition; 29.4–51.0% decreases in *GS1;1*-overexpressing plants and 22.7–35.1% decreases in *GS1;2*-overexpressing plants under four different nitrogen levels were observed (Table 2). In addition, the yield components (panicles/plant, filled grains/panicle, seed setting rate and thousand grains weight) were analyzed. During the panicles/plant analysis, compared to wildtype plants, 14.7%, 20.0% and 12.3% decreases were observed in *GS1;1*-overexpressing plants under the 0×N, 0.1×N and 5×N conditions, respectively; while 35.3% decrease was observed in *GS1;2*-overexpressing plants under the 0×N condition (Table 2). During the filled grains/panicle analysis, compared to wildtype plants, 37.4%, 32.7% and 19.7% decreases were observed in *GS1;1*-overexpressing plants under the 0.1×N, 1×N and 5×N conditions, respectively; while 30.1% and 30.5% decreases were observed in *GS1;2*-overexpressing plants under the 1×N and 5×N conditions, respectively (Table 2). For the seed setting rate analysis, 14.0%, 28.0% and 19.0% decreases were observed in *GS1;1*-overexpressing plants under the 0×N, 0.1×N and 1×N conditions, respectively; while 17.4% and 23.9% increases were observed in *GS1;2*-overexpressing plants under the 0×N and 0.1×N conditions, respectively; and 20.2% decrease was observed in *GS1;2*-overexpressing plants under the 1×N condition (Table 2). For the thousand grains weight analysis, 7.9% decrease was observed in *GS1;1*-overexpressing plants under the 0×N condition; while 7.7% and 10.3% decreases were observed in *GS1;2*-overexpressing plants under the 0×N and 5×N conditions, respectively (Table 2). These results suggested that the accumulated mRNA transcriptional levels of *GS1;1* and *GS1;2* affected the plant growth at the tillering and heading stages and also seriously affected productivity at the mature stage.

**Table 2:** The yield and its components in the *GS1;1*-, *GS1;2*-overexpressing plants (OX-*GS1;1*, OX-*GS1;2*) and wildtype plants (WT) under 0×N, 0.1×N, 1×N, 5×N conditions.

	Panicles/plant	Filled grains/panicle	Seed settingrate (%)	Thousand grainsweight (g)	yield(g/plant)
**0×N**					
WT	3.4±0.3 a	19.1±1.5 a	35.1±1.5 b	24.46±0.65 a	1.60±0.11 a
OX-*GS1;1*	2.9±0.1 b	17.0±1.5 a	30.2±0.3 c	22.52±0.33 b	1.13±0.07 b
OX-*GS1;2*	2.2±0.2 c	22.3±2.5 a	41.2±2.1 a	22.57±0.06 b	1.12±0.07 b
**0.1×N**					
WT	4.0±0.2 a	21.4±3.5 a	36.4±4.3 b	23.05±0.74 a	2.00±0.44 a
OX-*GS1;1*	3.2±0.2 b	13.4±3.0 b	26.2±1.6 c	22.23±0.72 a	0.97±0.29 b
OX-*GS1;2*	3.9±0.2 a	27.1±4.4 a	45.1±2.9 a	22.11±0.39 a	2.36±0.55 a
**1×N**					
WT	11.5±1.1 a	44.9±4.9 a	66.9±5.9 a	24.98±0.21 a	12.78±0.42 a
OX-*GS1;1*	9.9±0.7 a	30.2±0.3 b	54.2±3.9 b	24.73±0.92 a	7.41±0.74 c
OX-*GS1;2*	12.9±1.0 a	31.4±1.6 b	53.4±3.9 b	24.41±0.89 a	9.88±0.84 b
**5×N**					
WT	14.6±0.9 a	32.5±2.3 a	65.9±10.9 a	22.77±0.75 a	10.80±0.41 a
OX-*GS1;1*	12.8±0.3 b	26.1±2.0 b	59.0±9.2 a	22.26±0.16 a	7.20±0.75 b
OX-*GS1;2*	15.3±3.5 a	22.6±3.7 b	60.0±5.3 a	20.43±0.56 b	7.01±1.56 b

Values are mean ± SD from ten randomly selected plants. a, b, c indicate the significant difference at the level of P = 0.05.

### Nitrogen Uptake Assay of GS1-overexpressing Plants

**Figure 2:**
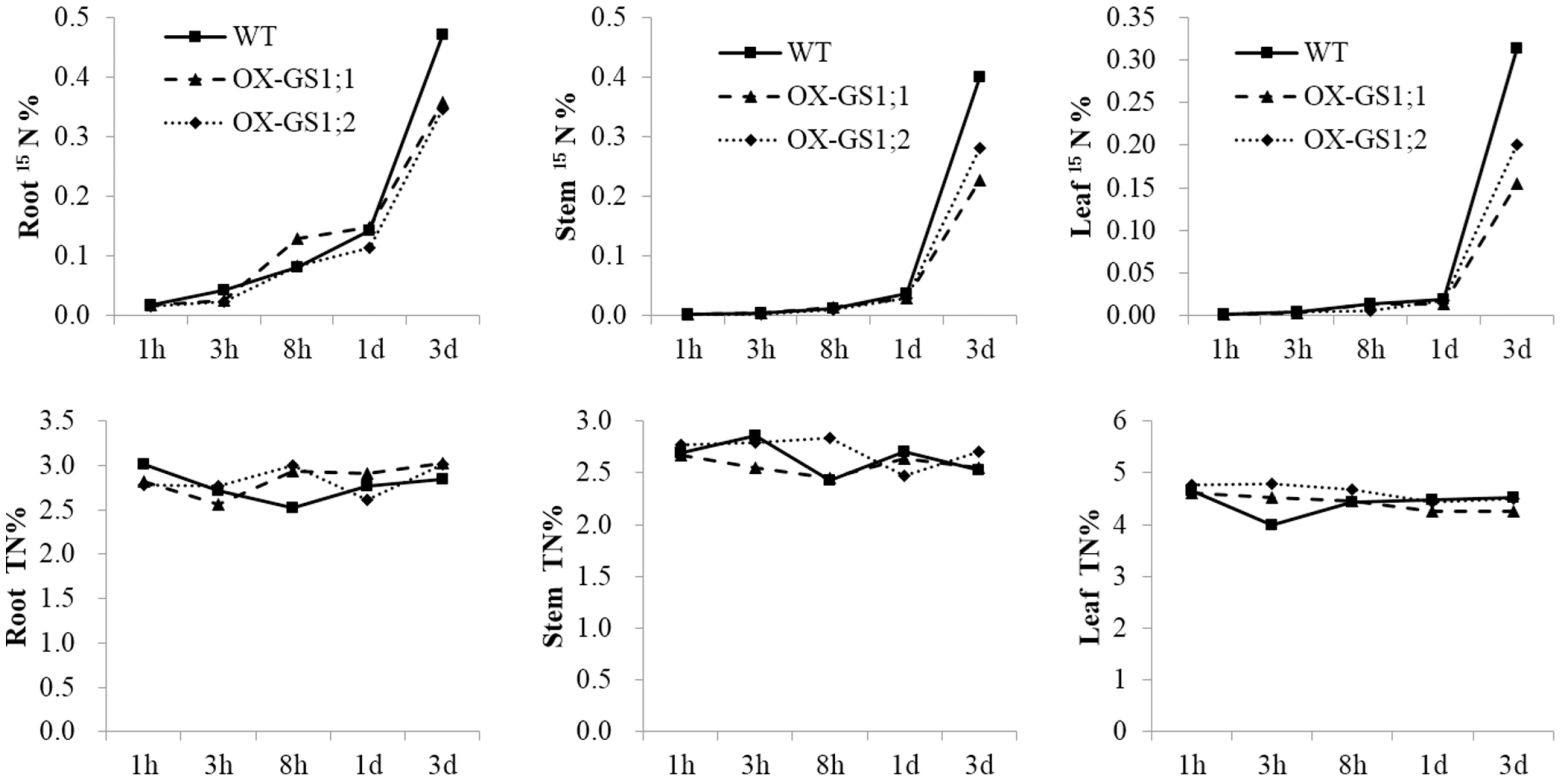
The ^15^N (^15^N%) and total nitrogen content (TN%) in the roots, stems and leaves of the *GS1;1*-, *GS1;2*-overexpressing plants (OX-*GS1;1*, OX-*GS1;2*) and wildtype plants (WT) at 1 h, 3 h, 8 h, 1 d and 3 d after NH_4_NO_3_ in the nutrient solution was replaced with ^15^NH_4_^15^NO_3_ during the tillering stage. Values are the means from six randomly mixed plant materials.

Because glutamine synthetase is the main enzyme involved in nitrogen assimilation, we analyzed the contents of ^15^N and total N in the root, stem and leaves of *GS1;1*-, *GS1;2*-overexpressing plants and wildtype plants using the ^15^N tracer assay at the tillering stage to test the differences in the nitrogen uptake ability in the root, and nitrogen transport ability from the root to the stem and from the stem to leaf between *GS1;1*-, *GS1;2*-overexpressing plants and wildtype plants. Our results showed that both *GS1;1*- and *GS1;2*-overexpressing plants demonstrated lower ^15^N contents in the root, stem and leaf, particularly at 3 d after NH_4_NO_3_ in the nutrient solution when refreshed by ^15^NH_4_^15^NO_3_ (Fig. 2). Interestingly, similar ^15^N contents in the root, while different ^15^N contents in the stem and leaf between *GS1;1*- and *GS1;2*-overexpressing plants were observed at 3 d after NH_4_NO_3_ in the nutrient solution when refreshed by ^15^NH_4_^15^NO_3_ (Fig. 2). Furthermore, we analyzed the total carbon and nitrogen contents in the root, stem and leaves of *GS1;1*-, *GS1;2*-overexpressing plants and wildtype plants under the 0×N, 0.1×N, 1×N and 5×N conditions at this stage. Due to the two independent assays, the plant materials were different from the materials used in the ^15^N tracer assay. Our results showed significant (P<0.05) decreases in the carbon/nitrogen ratio, specifically in the stem, in the *GS1;1*-, *GS1;2*-overexpressing plants when compared to wildtype plants. For the *GS1;1*-overexpressing plants, there were 22.3% and 9.6% decreases in the stem carbon/nitrogen ratio under the 0.1×N and 1×N conditions, respectively, which was caused by 22.1% and 9.4% higher stem nitrogen contents under the 0.1×N and 1×N conditions, respectively (Table 3). Similar results were also observed in *GS1;2*-overexpressing plants, in which there was a 9.9% decrease in the leaf carbon/nitrogen ratio under the 0×N condition, and 13.7% and 12.3% decreases in stem carbon/nitrogen ratio under the 1×N and 5×N conditions, respectively, which was caused by a 10.3% higher leaf nitrogen content under the 0×N condition, and 18.0% and 12.2% higher stem nitrogen contents under the 1×N and 5×N conditions, respectively (Table 3).

**Table 3:** The carbon content (C%), nitrogen content (N%) and carbon/nitrogen ratio (C/N) in the roots, stems and leaves of the *GS1;1*-, *GS1;2*-overexpressing plants (OX-*GS1;1*, OX-*GS1;2*) and wildtype plants (WT) at the tillering stage under 0×N, 0.1×N, 1×N, 5×N conditions.

	C%			N%			C/N		
	Root	Stem	Leaf	Root	Stem	Leaf	Root	Stem	Leaf
**0×N**									
WT	38.54±0.60 a	38.26±0.43 a	40.10±0.20 a	1.76±0.06 a	0.96±0.04 a	2.14±0.03 b	21.85±0.77 a	39.79±1.05 a	18.71±0.25 a
OX-*GS1;1*	38.22±0.41 a	37.74±0.21 ab	39.22±0.19 b	1.79±ND a	0.95±0.02 a	2.15±0.12 b	21.32±0.05 a	39.63±0.50 a	18.28±0.96 a
OX-*GS1;2*	38.71±0.80 a	36.72±0.46 b	39.68±0.33ab	1.75±0.13 a	1.05±0.11 a	2.36±0.13 a	22.21±1.74 a	35.10±3.36 a	16.85±0.81 b
**0.1×N**									
WT	38.47±0.07 a	38.88±0.10 a	40.52±0.16 a	2.10±0.14 a	1.40±0.11 b	2.91±0.18 a	18.34±1.17 a	27.80±2.20 a	13.94±0.81 a
OX-*GS1;1*	37.59±0.62 a	36.65±0.90 a	39.26±0.71 b	2.44±0.13 a	1.71±0.13 a	2.90±0.14 a	15.41±1.10 a	21.59±2.19 b	13.56±0.63 a
OX-*GS1;2*	37.34±0.60 a	37.66±0.64 a	40.38±0.38 ab	2.20±0.06 a	1.66±0.21 ab	3.16±0.11 a	16.94±0.31 a	22.93±3.16 ab	12.78±0.47 a
**1×N**									
WT	37.48±0.60 a	37.00±0.36 a	41.46±0.25 a	2.72±0.04 a	2.44±0.08 c	3.75±0.08 a	13.77±0.20 a	15.19±0.49 a	11.05±0.16 a
OX-*GS1;1*	37.82±0.01 a	36.59±0.34 a	40.79±0.66 a	2.85±ND a	2.67±0.06 b	3.80±0.16 a	13.27±ND a	13.73±0.20 b	10.75±0.28 a
OX-*GS1;2*	39.18±0.08 a	37.75±1.37 a	41.91±0.62 a	2.81±0.06 a	2.88±0.02 a	3.87±0.06 a	13.94±0.30 a	13.11±0.37 b	10.83±0.02 a
**5×N**									
WT	37.69±0.61 a	36.96±0.18 a	41.46±0.25 b	2.85±0.16 a	3.04±0.08 b	3.98±0.08 b	13.27±1.00 a	12.15±0.31 a	10.41±0.15 a
OX-*GS1;1*	ND	36.59±0.22 a	41.29±0.57 b	ND	3.43±0.35 ab	3.97±0.22 b	ND	10.75±1.18 ab	10.43±0.65 a
OX*-GS1;2*	ND	36.38±0.36 a	44.48±0.26 a	ND	3.41±0.14 a	4.28±0.12 a	ND	10.66±0.34 b	10.40±0.34 a

Values are mean ± SD from three biological replications using three randomly mixed plant materials. a, b, c indicate the significant differences at the level of P = 0.05. ND: no data.

### Soluble Proteins and Carbohydrates Determination of GS1-overexpressing Plants

**Figure 3:**
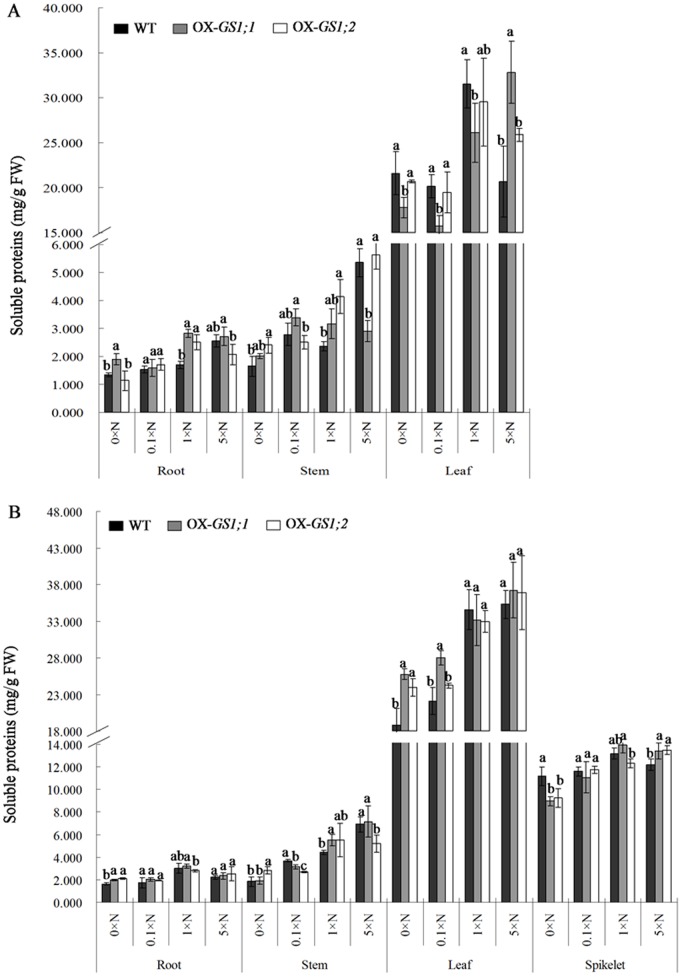
The concentration of soluble proteins in the roots, stems and leaves of the *GS1;1*-, *GS1;2*-overexpressing plants (OX-*GS1;1*, OX-*GS1;2*) and wildtype plants (WT) at the tillering stage (A) and the heading stage (B) under 0×N, 0.1×N, 1×N and 5×N conditions. Values are the mean ± SD from three biological replications using three randomly mixed plant materials. a, b, c indicate the significant difference at the level of P = 0.05.

**Figure 4:**
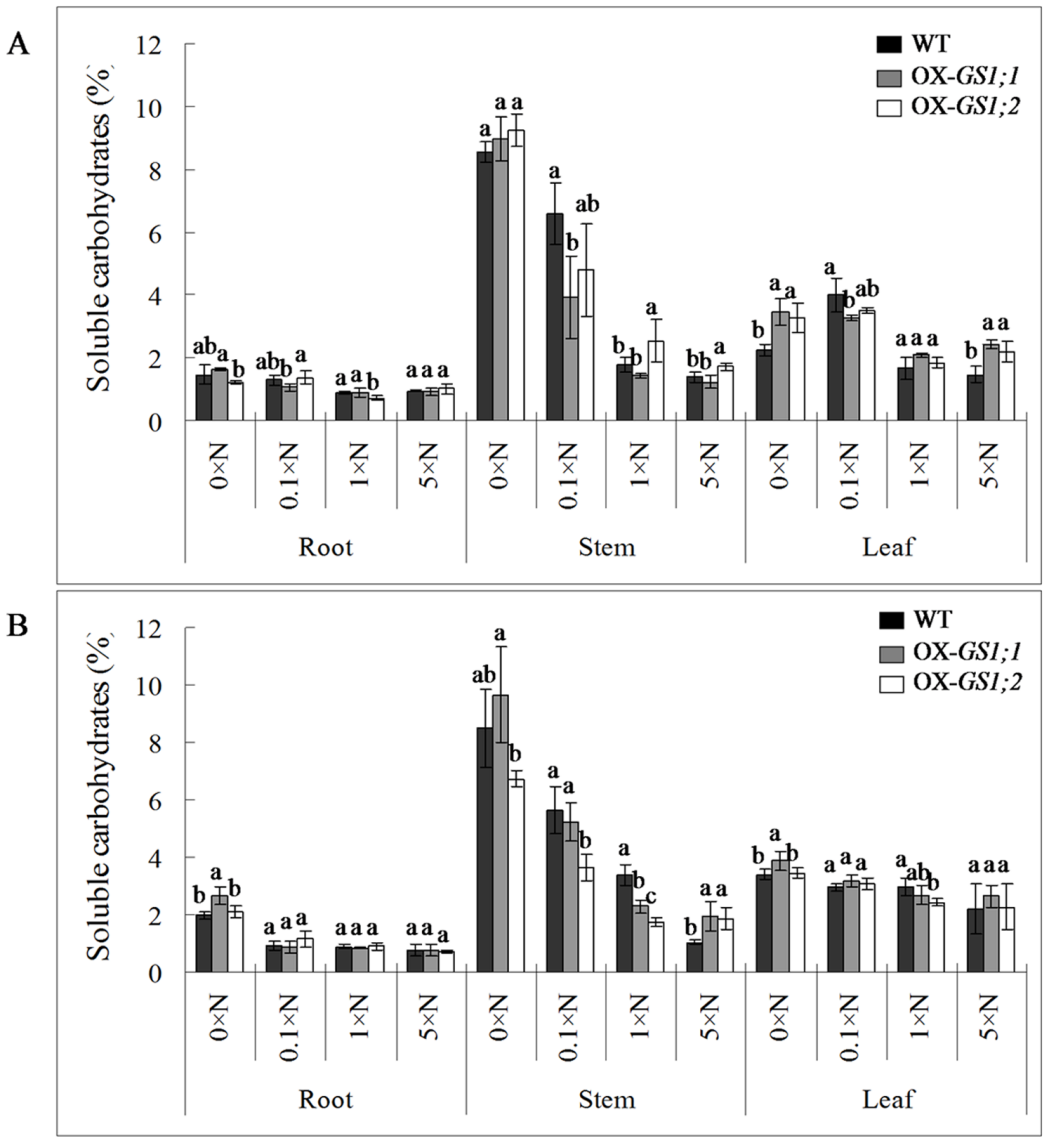
The concentration of soluble carbohydrates in the roots, stems and leaves of the *GS1;1*-, *GS1;2*-overexpressing plants (OX-*GS1;1*, OX-*GS1;2*) and wildtype plants (WT) at the tillering stage (A) and the heading stage (B) under 0×N, 0.1×N, 1×N and 5×N conditions. Values are the mean ± SD from three biological replications using three randomly mixed plant materials. a, b, c indicate the significant difference at the level of P = 0.05.

Because the nitrogen and carbon contents are altered by the over-expression of *GS1;1*, *GS1;2* genes as previously mentioned, we determined the concentrations of soluble proteins and carbohydrates in the root, stem and leaves of *GS1;1*-, *GS1;2*-overexpressing plants and wildtype plants at both the tillering and heading stages under the 0×N, 0.1×N, 1×N and 5×N conditions to evaluate the differences in the carbon and nitrogen metabolic status between *GS1;1-*, *GS1;2*-overexpressing plants and wildtype plants. Our results showed that most of the soluble proteins were present in the leaf, while most of the soluble carbohydrates were present in the stem. Interestingly, the soluble proteins increased from a low nitrogen level to a high nitrogen level, while the opposite pattern was observed in the soluble carbohydrates, which decreased from a low nitrogen level to a high nitrogen level at both the tillering and heading stages (Fig. 3, 4). Compared to wildtype plants, the concentration of soluble proteins was decreased by 11.7%, 15.1% and 9.8% and increased by 34.6% in *GS1;1*-overexpressing plants at the tillering stage. In contrast, the concentration of soluble proteins was increased by 15.5%, 13.1%, 1.2% and 6.2% in *GS1;1*-overexpressing plants at the heading stage under the 0×N, 0.1×N, 1×N and 5×N conditions, respectively (data not shown here). Similar results were found in the *GS1;2*-overexpressing plants, in which there were 1.5% and 3.1% decreases and 1.6% and 17.5% increases in soluble proteins at the tillering stage, and 14.2% and 3.9% increases, a 2.9% decrease, and a 2.5% increase in soluble proteins at the heading stage under the 0×N, 0.1×N, 1×N and 5×N conditions, respectively (data not shown here), were observed. Compared to wildtype plants, the concentration of soluble carbohydrates was increased by 14.6%, decreased by 30.5%, and increased by 1.8% and 20.6% in the *GS1;1*-overexpressing plants at the tillering stage, while a 16.6% increase, 2.4% and 12.8% decreases, and a 33.9% increase in *GS1;1*-overexpressing plants at the heading stage under the 0×N, 0.1×N, 1×N and 5×N conditions, respectively (data not shown here) were observed. Similar results were found in *GS1;2*-overexpressing plants, in which there was a 12.0% increase, a 18.5% decrease, and 16.9% and 29.0% increases in soluble carbohydrates at the tillering stage, while 11.4%, 17.2% and 21.1% decreases and a 21.2% increase in soluble carbohydrates at the heading stage under the 0×N, 0.1×N, 1×N and 5×N conditions, respectively (data not shown here) were observed.

Furthermore, there were many changes in the concentrations of the root, stem and leaf soluble proteins and carbohydrates in *GS1;1*-, *GS1;2*-overexpressing plants when compared to wildtype plants (Fig. 3, 4). During the analysis of soluble proteins, for *GS1;1*-overexpressing plants at the tillering stage, there were significant (P<0.05) increases in the root under the 0×N (42.1%) and 1×N (67.1%) conditions, in the leaf under the 5×N (58.9%) condition, and significant (P<0.05) decreases in the stem under the 5×N (45.6%) condition, in the leaf under the 0×N (17.6%), 0.1×N (21.6%) and 1×N (17.3%) conditions. However, at the heading stage, there were significant (P<0.05) increases in the root under the 0×N (22.2%) condition, in the stem under the 1×N (24.9%) condition, in the leaf under the 0×N (37.1%) and 0.1×N (26.7%) conditions, in the spikelet under the 5×N (9.6%) condition, and significant (P<0.05) decreases in the stem under the 0.1×N (14.3%) condition and in the spikelet under the 0×N (19.8%) condition (Fig. 3). For *GS1;2*-overexpressing plants, at the tillering stage, there were significant (P<0.05) increases in the root under the 1×N (47.9%) condition, in the stem under the 0×N (45.9%) and 1×N (74.9%) conditions, and in the leaf under the 5×N (25.2%) condition. However, at the heading stage, there were significant (P<0.05) increases in the root under the 0×N (30.1%) condition, in the stem under the 0×N (54.7%) condition, and in the leaf under the 0×N (27.5%) condition, and there were significant (P<0.05) decreases in the stem under the 0.1×N (26.4%) and 5×N (24.9%) conditions and in the spikelet under the 0×N (17.2%) condition (Fig. 3). During the soluble carbohydrates analysis, for *GS1;1*-overexpressing plants at the tillering stage, there were significant (P<0.05) increases in the root under the 0×N (53.7%) and 5×N (65.3%) conditions and significant (P<0.05) decreases in the stem under the 0.1×N (40.2%) condition and in the leaf under the 0.1×N (18.6%) condition. However, at the heading stage, there were significant (P<0.05) increases in the root under the 0×N (34.8%) condition, in the stem under the 5×N (89.0%) condition, and in the leaf under the 0×N (13.6%) condition, and there were significant (P<0.05) decreases in the stem under the 1×N (32.5%) condition (Fig. 3). For *GS1;2*-overexpressing plants at the tillering stage, there were significant (P<0.05) increases in the stem under the 1×N (42.7%) and 5×N (23.9%) conditions and in the leaf under the 0×N (45.2%) and 5×N (48.6%) conditions, and there was a significant (P<0.05) decrease in the root under the 1×N (19.4%) condition; at the heading stage, there was a significant (P<0.05) increase in the stem under the 5×N (81.5%) condition and significant (P<0.05) decreases in the stem under the 0.1×N (35.4%) and 1×N (49.1%) conditions and in the leaf under the 1×N (18.7%) condition (Fig. 4). These results suggested that over-expression of *GS1;1*, *GS1;2* not only changed the concentrations of soluble proteins and carbohydrates in transgenic plants but also altered their distribution in the root, stem, leaf and spikelet, which affected the carbon and nitrogen metabolic status.

### Metabolic Profiling Analysis in GS1-overexpressing Plants

**Figure 5:**
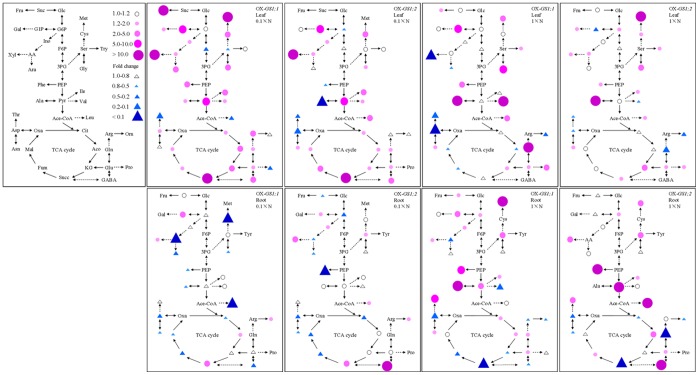
Fold change corresponding to the ratio of the concentration of individual metabolites involved in carbon and nitrogen metabolism in the *GS1;1*-, *GS1;2*-overexpressing plants relative to the wildtype plants for the leaves and roots at the tillering stage under 0.1×N and 1×N conditions. Glc, glucose; Suc, sucrose; Fru, Fructose; F6P, Frutose-6-P; G6P, Glucose-6-P; G1P, Glucose-1-P; Gal, galactose; Ino, Inositol; AA, Ascorbic acid; Ara, Arabinose; Xyl, Xylitol; 3PG, 3-P-glycerate; PEP, P-enolpyruvate; Pyr, Pyruvate; Ace-CoA, acetyl-CoA; Cit, Citrate; Aco, Aconitase; KG, Ketoglutarate; Succ, Succinate; Fum, Fumarate; Mal, Malate; Oxa, oxaloacetate; Glu, Glutamate; Gln, Glutamine; Arg, Arginine; Pro, Proline; Orn, Ornithine; GABA, Aminobutyric; Asp, Aspartate; Asn, Asparagine; Ile, Isoleucine; Met, Methionine; Thr, Threonine; Ala, Alanine; Val, Valine; Leu, Leucine; Phe, Phenylalanine; Try, Tryptophan; Ser, Serine; Gly, Glycine; Cys, Cysteine. Red dots indicate increased metabolites and blue triangles indicate decreased metabolites.

To further study the individual metabolite involved in the carbon and nitrogen metabolic pathway in detail, we analyzed the sugars, organic acids and free amino acids in the root and leaves of *GS1;1*-, *GS1;2*-overexpressing plants and wildtype plants at the tillering stage under the 0.1×N and 1×N conditions. As shown in Figure 5 and Figure S1, the fold change corresponds to the ratio of *GS1;1*-, *GS1;2*-overexpressing plants/wildtype plants calculated by the concentration of these individual metabolites. For the total sugars, total organic acids and total free amino acids, the results showed similar patterns between the *GS1;1*- and *GS1;2*-overexpressing plants, particularly under the 0.1×N condition. The total sugars, total organic acids and total free amino acids increased in the leaf and decreased in the roots of both *GS1;1*- and *GS1;2*-overexpressing plants under the 0.1×N condition (data not shown here). Under the 1×N condition, the total sugars, total organic acids and total free amino acids increased in the leaves of both *GS1;1*- and *GS1;2*-overexpressing plants, while opposite results were found in the root between the *GS1;1*- and *GS1;2*-overexpressing plants, in which the total sugars and total organic acids increased in the *GS1;1*-overexpressing plants and decreased in the *GS1;2*-overexpressing plants. In addition, the total free amino acids decreased in the *GS1;1*-overexpressing plants and increased in the *GS1;2*-overexpressing plants (data not shown here).

As shown in Figure 5 and Figure S1, which displayed the fold change of the individual sugars, organic acids and free amino acids in *GS1;1*-, *GS1;2*-overexpressing plants compared to wildtype plants, we observed alterations in these metabolites due to the over-expression of the *GS1;1*, *GS1;2* gene. In general, larger variations were found in the leaf compared to the root. In the leaf, larger variations of individual sugars and organic acids were found under the 0.1×N condition, while larger variations in the individual free amino acids were found under the 1×N condition (Fig. 5, Fig. S1). In the root, larger variations of these individual metabolites were found under the 1×N condition compared to the 0.1×N condition (Fig. 5, Fig. S1). In the leaves of *GS1;1*-overexpressing plants, when compared to wildtype plants, dramatic increases in fructose (>10.8-fold), xylitol (>179.4-fold), succinate (>70.4-fold) and methionine (>166.8-fold) and dramatic decreases in benzoic acid (<0.02-fold) were observed under the 0.1×N condition, In contrast, dramatic increases in glutamine (>12.7-fold), alanine (>39.4-fold) and valine (>31.4-fold) and dramatic decreases in xylitol (<0.01-fold) and aspartate (<0.08-fold) were observed under the 1×N condition (Fig. 5, Fig. S1). In the roots of *GS1;1*-overexpressing plants, when compared to wildtype plants, dramatic decreases in ascorbic acid (<0.02-fold), leucine (<0.001-fold) and cysteine (<0.01-fold) were observed under the 0.1×N condition, while dramatic increases in benzoic acid (>52.3-fold), methionine (>16.4-fold) and alanine (>89.7-fold) and a dramatic decrease in succinate (<0.09-fold) were observed under the 1×N condition (Fig. 5, Fig. S1). In the leaves of *GS1;2*-overexpressing plants, when compared to wildtype plants, dramatic increases in xylitol (>202.8-fold) and succinate (>391.1-fold), and dramatic decreases in benzoic acid (<0.08-fold) and alanine (<0.01-fold) were observed under the 0.1×N condition, while dramatic increases in methionine (>48.1-fold) and alanine (>11.3-fold) were observed under the 1×N condition (Fig. 5, Fig. S1). In the roots of *GS1;2*-overexpressing plants, when compared to wildtype plants, a dramatic increase in aminobutyric acid (>93.8-fold) and a dramatic decrease in phenylalanine (<0.04-fold) were observed under the 0.1×N condition, while dramatic increases in pyruvate (>708.3-fold), aminobutyric acid (>69.9-fold), leucine (>14.4-fold) and phenylalanine (>14.8-fold) and dramatic decreases in succinate (<0.04-fold) and glutamine (<0.03-fold) were observed under the 1×N condition (Fig. 5, Fig. S1).

### Gene Expression Analysis in GS1-overexpressing Plants

**Figure 6:**
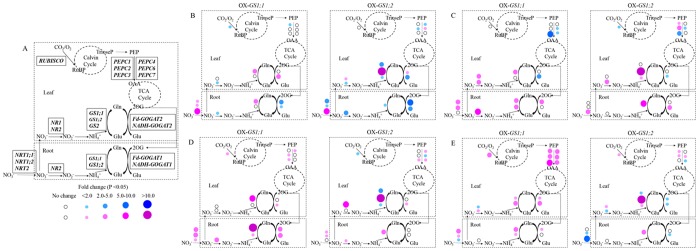
Fold change corresponding to the ratio of the gene expression level in the *GS1;1*-, *GS1;2*-overexpressing plants relative to the wildtype plants. (A) Diagrammatic representation of the key genes involved in the carbon and nitrogen metabolic pathway in rice plants. NRT, nitrate transporter; NR, nitrate reductase; GS, glutamine synthetase; GOGAT, glutamate synthase; RUBISCO, ribulose-1,5-bisphosphate carboxylase/oxygenase; PEPC, phosphoenolpyruvate carboxylase. Prominent changes in the gene expression level in the *GS1;1*-, *GS1;2*-overexpressed plants compared to wild type plants at the tillering stage under 0×N (B), 0.1×N (C), 1×N (D) and 5×N (E) conditions. Red and blue dots indicate up- and down-regulated genes, respectively.

To analyze the effect of higher *GS1;1*, *GS1;2* mRNA transcriptional levels on the expression patterns of key genes involved in the carbon and nitrogen metabolic pathway, the expression level of genes encoding NRT (nitrate transporter), NR (nitrate reductase), GS (glutamine synthetase), GOGAT (glutamate synthase), RUBISCO (ribulose-1,5-bisphosphate carboxylase/oxygenase) and PEPC (phosphoenolpyruvate carboxylase) were analyzed using q-RT PCR. These genes are displayed in the carbon and nitrogen metabolic pathway in rice plants (Fig. 6A). Table S2 lists the fold change corresponding to the ratio of *GS1;1-*, *GS1;2*-overexpressing plants/wildtype plants calculated by the relative gene expression level in the root and leaf at the tillering stage under the 0×N, 0.1×N, 1×N and 5×N conditions. Our results showed the distinct expression patterns of these genes displayed between the *GS1;1-* and *GS1;2*-overexpressing plants under different nitrogen levels.

Under the 0×N condition, compared to wildtype plants, the expression levels of *NR2*, *Fd-GOGAT1* and *NADH-GOGAT1* significantly (P<0.05) decreased in the roots of *GS1;1*-overexpressing plants, and the expression levels of *NRT1;1*, *NRT1;1*, *NRT2* and *GS1;1* were significantly (P<0.01) increased. However, in the leaves of *GS1;1*-overexpressing plants, the expression levels of *NR2*, *RUBISCO* and *PEPC1* significantly (P<0.05) decreased, and the expression levels of *GS1;1* and *NADH-GOGAT2* significantly (P<0.01) increased (Fig. 6B, Table S2). In the roots of *GS1;2*-overexpressing plants, the expression levels of most of the genes (*NRT1;1*, *NRT1;2*, *NR2*, *GS1;2*, *Fd-GOGAT1* and *NADH-GOGAT1*) were significantly (P<0.05) decreased, except one gene (*NRT2*), which showed a significant (P<0.01) increase in its expression level. In contrast, in the leaves of *GS1;2*-overexpressing plants, the expression levels of *NR2*, *GS1;1*, *GS2*, *NADH-GOGAT2*, *PEPC2* and *PEPC4* significantly (P<0.05) decreased, and the expression levels of *NR1*, *GS1;2*, *Fd-GOGAT2* and *PEPC6* significantly (P<0.05) increased (Fig. 6B, Table S2).

Under the 0.1×N condition, compared to wildtype plants, in the roots of *GS1;1*-overexpressing plants, most of the genes (*NRT1;1*, *NRT1;2*, *NR2*, *GS1;1*, *GS1;2* and *Fd-GOGAT1*) were significantly (P<0.01) induced, while in the leaves of *GS1;1*-overexpressing plants, the expression levels of *NADH-GOGAT2*, *PEPC3*, *PEPC6* and *PEPC7* were significantly (P<0.01) decreased, and the expression levels of *NR1*, *NR2* and *GS1;1* were significantly (P<0.05) increased (Fig. 6C, Table S2). Similar expression patterns demonstrated that in *GS1;2*-overexpressing plants, *NRT2*, *GS1;2* and *NADH-GOGAT1* were significantly (P<0.01) induced in the root, and the expression levels of *NADH-GOGAT2*, *RUBISCO*, *PEPC3* and *PEPC6* significantly (P<0.05) decreased and the expression levels of *NR2*, *GS1;2*, *GS2, PEPC1* and *PEPC2* significantly (P<0.05) increased in the leaf (Fig. 6C, Table S2).

Under the 1×N condition, compared to wildtype plants, most of the genes were significantly (P<0.05) induced in *GS1;1*-overexpressing plants, such as *NRT1;1*, *NRT1;2*, *NRT2*, *NR2*, *GS1;1*, *GS1;2*, *Fd-GOGAT1* and *NADH-GOGAT1* genes in the root, and *GS1;1, NADH-GOGAT2*, *RUBISCO*, *PEPC2*, *PEPC3* and *PEPC4* genes in the leaf (Fig. 6D, Table S2). Similarly, in *GS1;2*-overexpressing plants, most of the genes were significantly (P<0.05) induced (including *NRT1;1*, *NRT1;2*, *NR2* and *GS1;2* genes in the root, *NR1*, *GS1;2*, *NADH-GOGAT2*, *PEPC3*, *PEPC4* and *PEPC7* genes in the leaf), except for the *GS1;1*, *GS2*, *RUBISCO* and *PEPC6* genes, which were significantly (P<0.05) decreased in the leaf (Fig. 6D, Table S2).

Under the 5×N condition, compared to wildtype plants, most of the genes were significantly (P<0.05) induced in *GS1;1*-overexpressing plants (such as *NRT1;1*, *NRT1;2*, *GS1;1*, *GS1;2* and *Fd-GOGAT1* genes in the root, *NR2*, *GS1;1*, *GS2*, *Fd-GOGAT2*, *NADH-GOGAT2*, *RUBISCO*, *PEPC1*, *PEPC2*, *PEPC3*, *PEPC4*, *PEPC6* and *PEPC7* genes in the leaf), except for the *NRT2* gene in the root and the *NR1* gene in the leaf, which were significantly (P<0.05) decreased (Fig. 6E, Table S2). Different expression patterns were found in *GS1;2*-overexpressing plants, in which the expression levels of *NRT1;2*, *NRT2* and *NADH-GOGAT1* were significantly (P<0.05) decreased and the expression level of *GS1;2* was significantly (P<0.01) increased in the root. However, in the leaf, the expression levels of *NR2*, *GS1;1*, *Fd-GOGAT2*, *NADH-GOGAT2* and *PEPC7* were significantly (P<0.05) decreased, and the expression levels of *NR1*, *GS1;2*, *GS2*, *RUBISCO*, *PEPC1*, *PEPC2* and *PEPC6* were significantly (P<0.05) increased (Fig. 6E, Table S2).

## Discussion

### Cytosolic GS1 Genes Play Important Role in Plant Growth and Grain Filling

In higher plants, GS/GOGAT cycle is the first step of incorporation of inorganic nitrogen into organic nitrogenous compounds, which is a major checkpoint for controlling nitrogen assimilation. Because of this important function of GS enzyme in plant nitrogen metabolism, particular attention has been devoted to studies on *GS* transformation in higher plants, which is expected to be a good molecular method used to analyze gene functions and a good strategy to improve nitrogen use efficiency. In our previous study, the full length cDNAs of cytosolic *GS1;1* and *GS1;2* in rice were constructed into *pCAMBIA* 1301S vector which driven by the *CaMV* 35S promoter; and the *GS1*-overexpressing transgenic plants were obtained. The fresh weight and dry weight of *GS1;1*-, *GS1;2*-overexpressing plants were not significantly different than those of wildtype plants at the seedling stage [43]. However, the *GS1;1*-, *GS1;2*-overexpressing plants showed significantly decreased plant height and root length, shoot dry weight and root dry weight at both the tillering stage and heading stage when compared to the wildtype plants. These results indicated a high expression level of cytosolic *GS1* gene was negatively correlated with plant biomass. Similar results were also observed in *GS1*-overexpressing Lotus japonicus and Pea, which exhibited negative effect on plant biomass [35], [36]. In addition, several studies highlighted the importance of cytosolic *GS1* genes in determining grain filling in cereal crops. For example, positive correlations were showed between grain number/size and cytosolic GS protein content/GS activity in rice [24], [52], [53], maize [38], [54], [65] wheat [34]. However, in our study, a negative correlation was observed between the cytosolic *GS1* gene expreesion level and the grain filling in *GS1;1*-, *GS1;2*-overexpressing plants. Compared to the wildtype plants, the yields of *GS1;1*-, *GS1;2*-overexpressing plants were significantly declined. Similar results were reported in the previous study that *GS1;1*-, *GS1;2*-overexpressing plants showed decreased yield and amino acids concentrations when grown under the low nitrogen field [43].

### Stable Carbon/nitrogen Ratio Plays an Important Role in Plant Development

In higher plants, carbon and nitrogen are crucial for routine and fundamental cellular activities. Plant growth and development are highly dependent on the interaction between carbon and nitrogen metabolism. The coordination and optimal functioning of the metabolic pathways for nitrogen and carbon assimilation in plants are critical in determining plant growth, biomass accumulation and, ultimately, yield [5], [7], [8], [10]. Due to developmental changes and environmental demands on carbon and nitrogen resources, plants have evolved as a highly sophisticated and complex sensory system to regulate carbon and nitrogen assimilation, metabolism and transport [10]. Studies at the molecular level have revealed an even higher complexity, with multiple levels of regulation, including post-transcriptional control by microRNAs [55]–[58]. Recent reports have also shown that the adaptation of maize source leaf metabolism to stress is related to disturbances in the carbon, nitrogen and phosphorus balance [59]. In the present study, significant (P<0.05) decrease of the carbon/nitrogen ratio were shown in *GS1;1*-, *GS1;2*-overexpressing plants, particularly in the stem. Furthermore, the concentrations of soluble proteins and carbohydrates changed their distributions in various organs, such as the root, stem, leaf and spikelet, which affected the carbon and nitrogen metabolic status. In addition, variations in the leaf SPAD value, photosynthetic parameters, individual metabolites and gene expression levels indicated an imbalance of carbon-nitrogen metabolism, which may cause the poor growth and yield in the *GS1;1*-, *GS1;2*-overexpressing plants. An imbalance in levels of sugars, amino acids and metabolites in the tricarboxylic acid cycle was also reported in the rice *OsGS1;1* mutant by Kusano et al., which revealed a crucial function of *OsGS1;1* in coordinating the global metabolic network in rice plants [37]. Previous studies have reported similar results in which the carbon to nitrogen ratio, rather than the carbohydrate status alone, plays a predominant role in regulating various aspects of *Arabidopsis* seedling growth, including the cotyledon size, fresh weight, chlorophyll and anthocyanin content, storage reserve mobilization and photosynthetic gene expression [9].

### GS1;1 and GS1;2 Genes have Different Functions in Rice Carbon and Nitrogen Metabolism

In higher plants, multiple homologous but distinct genes were found for cytosolic GS1 [14], [60]–[65], and three members have been identified in rice (*Oryza sativa*; *OsGS1;1*, *OsGS1;2* and *OsGS1;3*;) [2], [20], [24]. *OsGS1;1* was expressed in all organs (i.e., root, leaf blade, leaf sheath, and spikelet), with a higher expression in the leaf blade and shoot phloem during the vegetative stage and thus functions in translocating nitrogenous compounds to the developing sink tissues [24]. *OsGS1;2* transcripts were also detected in all organs, with a higher expression in the root at the seedling stage, and this protein mainly functions in primary NH_4_^+^ assimilation [2]. Although similar results of growth phenotype, yield and carbon/nitrogen ratio were found in both *GS1;1*- and *GS1;2*-overexpressing plants in this study, the different exhibitions of the leaf SPAD value, photosynthetic parameters, soluble proteins and carbohydrates, metabolites and gene expression levels were observed between the *GS1;1*- and *GS1;2*-overexpressing plants, suggesting different roles of *GS1;1* and *GS1;2* genes in rice nitrogen metabolism. In addition, different profiles of individual metabolites and gene expression levels were found between *GS1;1*- and *GS1;2*-overexpressing plants, particularly when sufficient nitrogen was applied in the environment. Our previous study also suggested different functions between the *GS1;1* and *GS1;2* genes in rice nitrogen metabolism and abiotic stress responses. Basta resistance was observed in *GS1;2*-overexpressing rice plants at three developmental stages (seed germination, two-leaf seedling, and mature plant stages) [43]. However, Basta resistance was not observed in *GS1;1*-overexpressing rice plants at any developmental stage [43]. In addition, analysis of *gln1-3* and *gln1-4* mutants in maize also indicated distinct roles of cytosolic *glutamine synthetase* genes in which the *gln1-4* mutant exhibited a reduced kernel size and the *gln1-3* mutant exhibited a reduced kernel number [38].

## Conclusion

In this study, we systematically analyzed the differences in the growth phenotype, carbon-nitrogen metabolic status and gene expression profile between *GS1;1-, GS1;2*-overexpressing rice plants and wildtype plants. From the results, we found that significant alterations in the carbon and nitrogen metabolism were displayed by the overexpressing of *GS1;1* or *GS1;2* in the transgenic plants. For example, under the 1×N condition, compared to wildtype plants, most of the carbon-nitrogen metabolic genes were significantly (P<0.05) induced in *GS1;1*-overexpressing plants (*NRT1;1*, *NRT1;2*, *NRT2*, *NR2*, *GS1;1*, *GS1;2*, *Fd-GOGAT1* and *NADH-GOGAT1* genes in the roots, and *GS1;1, NADH-GOGAT2*, *RUBISCO*, *PEPC2*, *PEPC3* and *PEPC4* genes in the leaves), which directly resulted the increasing of total sugars, total organic acids and total free amino acids in the leaves, the increasing of total sugars and total organic acids and the decreasing of the total free amino acids in the roots. These changes of metabolites caused the decline of soluble proteins and the increase of soluble carbohydrates in the *GS1;1*-overexpressing plants. While in the *GS1;2*-overexpressing plants, most of the genes were significantly (P<0.05) induced (including *NRT1;1*, *NRT1;2*, *NR2* and *GS1;2* genes in the roots, *NR1*, *GS1;2*, *NADH-GOGAT2*, *PEPC3*, *PEPC4* and *PEPC7* genes in the leaves), except for the *GS1;1*, *GS2*, *RUBISCO* and *PEPC6* genes, which were significantly (P<0.05) decreased in the leaves. This type of gene expression profile directly resulted the increasing of total sugars, total organic acids and total free amino acids in the leaves, the decreasing of total sugars and total organic acids and the increasing of the total free amino acids in the roots of *GS1;2*-overexpressing plants. These changes of metabolites caused the increases of both soluble proteins and carbohydrates in the *GS1;2*-overexpressing plants. In addition, the significantly (P<0.05) decreased carbon/nitrogen ratio was observed in the stem of both *GS1;1*- and *GS1;2*-overexpressing plants. We concluded that the imbalance in carbon and nitrogen metabolic status may be the main reason for the decreased growth and yield in *GS1;1*-, *GS1;2*-overexpressing plants.

## Supporting Information

Figure S1 Fold change corresponds to the ratio of the concentration of individual metabolites involved in carbon and nitrogen metabolism in the *GS1;1*-, *GS1;2*-overexpressing plants relative to the wildtype plants for the leaves (A) and roots (B) at the tillering stage. Pi, phosphate; Suc (in the group of sugars), sucrose; Fru, Fructose; F6P, Frutose-6-P; G6P, Glucose-6-P; G1P, Glucose-1-P; Ara, Arabinose; Xyl, Xylitol; Ino, Inositol; AA, Ascorbic acid; GA, Glutaric acid; BA, Benzoic acid; Pyr, Pyruvate; Cit, Citrate; KG, Ketoglutarate; Suc (in the group of organic acids), Succinate; Fum, Fumarate; Mal, Malate; Aco, Aconitase; Gal, galactose; Glu, Glutamate; Gln, Glutamine; Arg, Arginine; Pro, Proline; Orn, Ornithine; GABA, Aminobutyric; Asp, Aspartate; Asn, Asparagine; Ile, Isoleucine; Met, Methionine; Thr, Threonine; Ala, Alanine; Val, Valine; Leu, Leucine; Phe, Phenylalanine; Try, Tryptophan; Ser, Serine; Gly, Glycine; Cys, Cysteine.
(DOC)

Table S1 Primer sequences of the key genes involved in the carbon and nitrogen metabolism used in qRT-PCR.
(DOC)

Table S2 Fold change corresponding to the ratio of the gene expression level in the *GS1;1-, GS1;2*-overexpressing plants relative to the wildtype plants for the roots and leaves at the tillering stage under the 0×N, 0.1×N, 1×N, 5×N conditions.
(DOC)
