# Supplementary material for: Accumulated Expression Level of Cytosolic Glutamine Synthetase 1 Gene (OsGS1;1 or OsGS1;2) Alter Plant Development and the Carbon-Nitrogen Metabolic Status in Rice
Source: PLoS One. 2014 Apr 17;9(4):e95581. doi: 10.1371/journal.pone.0095581 (PMC3990726; doi:10.1371/journal.pone.0095581)
Supplement: Figure S1 — Fold change corresponds to the ratio of the concentration of individual metabolites involved in carbon and nitrogen metabolism in the GS1;1-, GS1;2-overexpressing plants relative to the wildtype plants for the leaves (A) and roots (B) at the tillering stage. Pi, phosphate; Suc (in the group of sugars), sucrose; Fru, Fructose; F6P, Frutose-6-P; G6P, Glucose-6-P; G1P, Glucose-1-P; Ara, Arabinose; Xyl, Xylitol; Ino, Inositol; AA, Ascorbic acid; GA, Glutaric acid; BA, Benzoic acid; Pyr, Pyruvate; Cit, Citrate; KG, Ketoglutarate; Suc (in the group of organic acids), Succinate; Fum, Fumarate; Mal, Malate; Aco, Aconitase; Gal, galactose; Glu, Glutamate; Gln, Glutamine; Arg, Arginine; Pro, Proline; Orn, Ornithine; GABA, Aminobutyric; Asp, Aspartate; Asn, Asparagine; Ile, Isoleucine; Met, Methionine; Thr, Threonine; Ala, Alanine; Val, Valine; Leu, Leucine; Phe, Phenylalanine; Try, Tryptophan; Ser, Serine; Gly, Glycine; Cys, Cysteine. (DOC) [file pone.0095581.s001.doc]

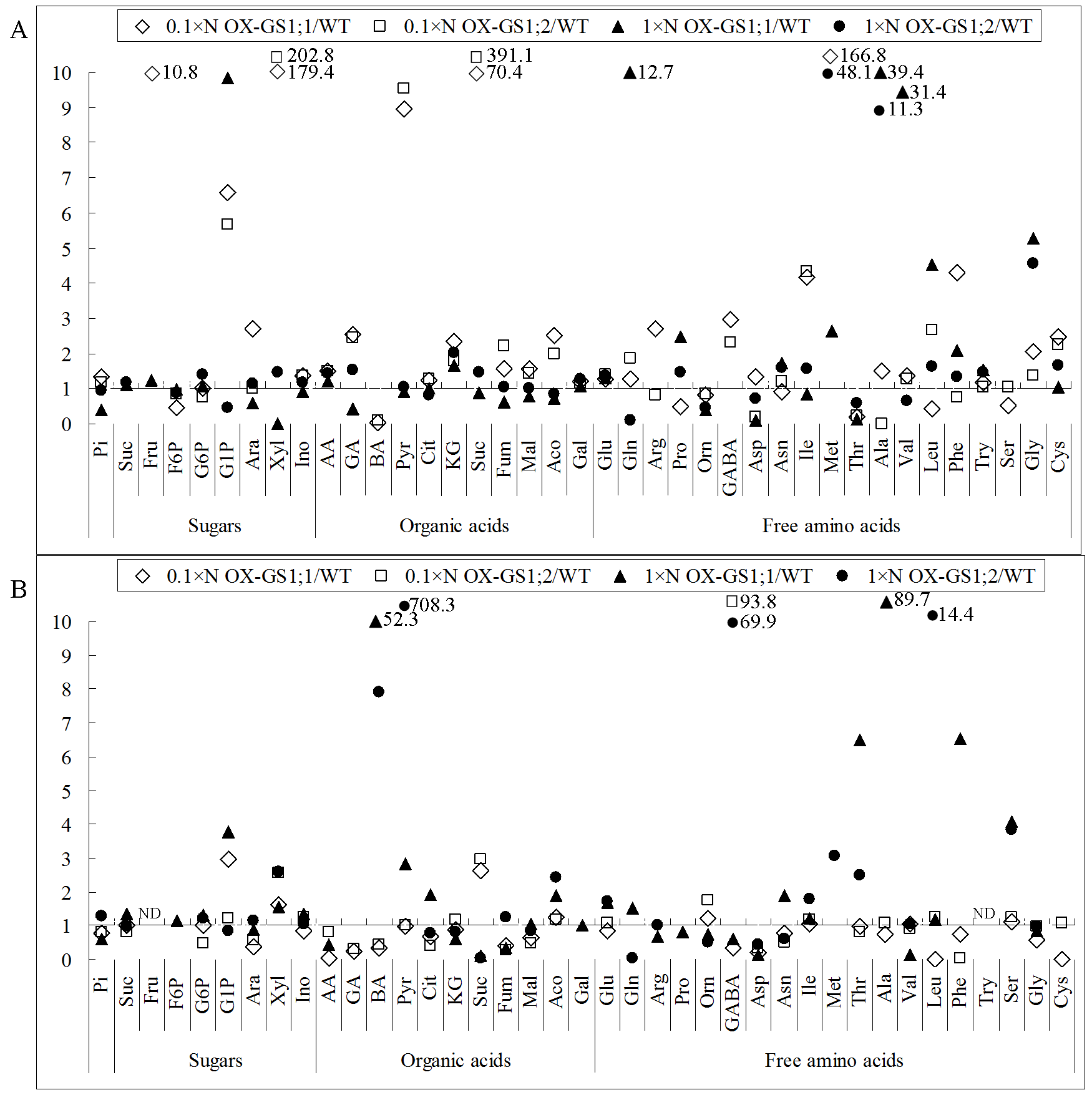


**Supplementary Fig. S1** Fold change corresponds to the ratio of the concentration of individual metabolites involved in carbon and nitrogen metabolism in the *GS1;1*-, *GS1;2*-overexpressed plants relative to the wild type plants for the leaves (A) and roots (B) at the tillering stage. Pi, phosphate; Suc (in the group of sugars), sucrose; Fru, Fructose; F6P, Frutose-6-P; G6P, Glucose-6-P; G1P, Glucose-1-P; Ara, Arabinose; Xyl, Xylitol; Ino, Inositol; AA, Ascorbic acid; GA, Glutaric acid; BA, Benzoic acid; Pyr, Pyruvate; Cit, Citrate; KG, Ketoglutarate; Suc (in the group of organic acids), Succinate; Fum, Fumarate; Mal, Malate; Aco, Aconitase; Gal, galactose; Glu, Glutamate; Gln, Glutamine; Arg, Arginine; Pro, Proline; Orn, Ornithine; GABA, Aminobutyric; Asp, Aspartate; Asn, Asparagine; Ile, Isoleucine; Met, Methionine; Thr, Threonine; Ala, Alanine; Val, Valine; Leu, Leucine; Phe, Phenylalanine; Try, Tryptophan; Ser, Serine; Gly, Glycine; Cys, Cysteine.
