# Supplementary material for: Accumulated Expression Level of Cytosolic Glutamine Synthetase 1 Gene (OsGS1;1 or OsGS1;2) Alter Plant Development and the Carbon-Nitrogen Metabolic Status in Rice
Source: PLoS One. 2014 Apr 17;9(4):e95581. doi: 10.1371/journal.pone.0095581 (PMC3990726; doi:10.1371/journal.pone.0095581)
Supplement: Table S1 — Primer sequences of the key genes involved in the carbon and nitrogen metabolism used in qRT-PCR. (DOC) [file pone.0095581.s002.doc]

**Supplementary Table S1**: Primer sequences of the key genes involved in the carbon and nitrogen metabolism used in qRT-PCR.

| Gene name | cDNA accession NO. | Primer sequence |
| --- | --- | --- |
| *NRT1;1* | AK066920 | F: CCTCGCAAGTGACCCTTGAAT |
|  |  | R: CGATGGCTAATGAGGAACCCTT |
| *NRT1;2* | AK101480 | F: GAACATGCGGATCATGTCGTT |
|  |  | R: CGATCACGGAGCTGTACATGAG |
| *NRT2* | AK109733 | F: TTCGCGAACCCGCATATGA |
|  |  | R: GTTGAGGTTGTCGCGGATGAT |
| *NR1* | AK102178 | F: ACTACCATTACCGCGACAACC |
|  |  | R: CTCGTTTATCATGTACTCCGGC |
| *NR2* | AK121810 | F: AGCTGAACGTGAACTCGGTGA |
|  |  | R: AGGCGTATCCCTTCATGGTGT |
| *GS1;1* | AK109397 | F: GAGTCGTCGTCTCATTTGACCC |
|  |  | R: GTAGCCACCATCGTTCCTCATC |
| *GS1;2* | AK243037 | F: TTTTCAAGGACCCGTTCAGGA |
|  |  | R: CGGCACTGTGCCTCTTGTTAGT |
| *GS1;3* | AK099290 | F: TCAAGCCATCTTCAGAGACCCA |
|  |  | R: TACCGGTTGTTCGTCGGAATC |
| *GS2* | AK063706 | F: AGGATCGGACAAATCGTTTGG |
|  |  | R: GCATGACCTCTCCATTTGTTCC |
| *Fd-GOGAT1* | AK102025 | F: AAATGCCTCTTTGCAAGGCC |
|  |  | R: GACTGTGAG CCCCATCCAAATA |
| *Fd-GOGAT2* | AK068130 | F: CCGATGCGATTGAGAATGAGA |
|  |  | R: CTTCTTGGCAATGACACCTGC |
| *NADH-GOGAT1* | AK105755 | F: TGCTTGAGAGAATGGCGCA |
|  |  | R: AACCCAGCATCCTTTGTCACC |
| *NADH-GOGAT2* | AK070485 | F: GGTTGTCATTGGTGGTGGAGA |
|  |  | R: TGGTGGCTCTGGCAAAAGTT |
| *RUBISCO* | AK243615 | F: AGGCTTCAAATTGCCGTTGA |
|  |  | R: TCTAGGCCATCCAGTTCCTCCT |
| *PEPC1* | AK100688 | F: ACATTCCGTGTTGCTGCAGAG |
|  |  | R: TGCAACAGTTCAACCGCTAGG |
| *PEPC2* | AK066635 | F: CAGAAGCACGCAAGCATTAGG |
|  |  | R: CGCGAGAATCTCTCTCTGAAGG |
| *PEPC3* | AK101274 | F: ACCGGTCCATTGTCTTCCAAG |
|  |  | R: CGTTTTGATGGCCTACTTCCAA |
| *PEPC4* | AK065425 | F: TGGATGAGATGGCTGTTGTGG |
|  |  | R: TTCTGTCTCAGGTGTTGCCGA |
| *PEPC6* | AK073703 | F: ATGTCTGCCAGGCTTACACGAT |
|  |  | R: CGGCTTAGACCAGTCCATGATC |
| *PEPC7* | AK242583 | F: GAGTATTTCCGCCTTGCAACAC |
|  |  | R: ACGGAGTGATTCAATGCCTCC |
| *ACTIN* | AK070531 | F: GACAATGGAACCGGAATGGTC |
|  |  | R: CCCAACCATAACGCCTGTATGT |
