# Supplementary material for: Accumulated Expression Level of Cytosolic Glutamine Synthetase 1 Gene (OsGS1;1 or OsGS1;2) Alter Plant Development and the Carbon-Nitrogen Metabolic Status in Rice
Source: PLoS One. 2014 Apr 17;9(4):e95581. doi: 10.1371/journal.pone.0095581 (PMC3990726; doi:10.1371/journal.pone.0095581)
Supplement: Table S2 — Fold change corresponding to the ratio of the gene expression level in the GS1;1-, GS1;2-overexpressing plants relative to the wildtype plants for the roots and leaves at the tillering stage under the 0×N, 0.1×N, 1×N, 5×N conditions. (DOC) [file pone.0095581.s003.doc]

**Supplementary Table S2**: Fold change corresponding to the ratio of the gene expression level in the *GS1;1-, GS1;2*-overexpressed plants relative to the wild type plants for the roots and leaves at the tillering stage under 0×N, 0.1×N, 1×N, 5×N conditions.

|  | 0×N | | | | |  | 0.1×N | | | | |
| --- | --- | --- | --- | --- | --- | --- | --- | --- | --- | --- | --- |
|  | Root | |  | Leaf | |  | Root | |  | Leaf | |
|  | OX-*GS1;1* | OX-*GS1;2* |  | OX-*GS1;1* | OX-*GS1;2* |  | OX-*GS1;1* | OX-*GS1;2* |  | OX-*GS1;1* | OX-*GS1;2* |
| *NRT1;1* | 1.58** | 0.29** |  | 2.05** | 0.46** |  | 3.55** | 1.12 |  | 1.06 | 2.60** |
| *NRT1;2* | 1.72** | 0.52** |  | 2.68** | 0.28** |  | 4.71** | 1.70 |  | 0.83 | 1.09 |
| *NRT2* | 5.95** | 6.38** |  | 9.00** | 0.78 |  | 0.74 | 2.96** |  | 1.61** | 0.98 |
| *NR1* | 0.84 | 3.12** |  | 0.85 | 1.75** |  | 0.51* | 0.08** |  | 2.26* | 0.92 |
| *NR2* | 0.53* | 0.22** |  | 0.84* | 0.85* |  | 5.53** | 1.04 |  | 3.48** | 2.38* |
| *GS1;1* | 2.26** | 0.09** |  | 2.21** | 0.43** |  | 3.83** | 0.91 |  | 4.56** | 1.18 |
| *GS1;2* | 0.83 | 0.64* |  | 1.87** | 79.94** |  | 4.83** | 8.92** |  | 0.86 | 113.73** |
| *GS1;3* | 0.99 | 0.28** |  | 2.29** | 0.25** |  | 2.00** | 3.03** |  | 1.71* | 9.37** |
| *GS2* | 0.71* | 0.22** |  | 0.90 | 0.68** |  | 58.09** | 2.65 |  | 0.95 | 1.21* |
| *Fd-GOGAT1* | 0.36** | 0.18** |  | 1.28* | 0.99 |  | 2.23** | 0.86 |  | 1.50** | 1.03 |
| *Fd-GOGAT2* | 0.30** | 0.27** |  | 0.97 | 1.50** |  | 4.10** | 1.12 |  | 0.77 | 0.90 |
| *NADH-GOGAT1* | 0.62** | 0.28** |  | 1.42** | 0.65** |  | 1.20 | 2.20** |  | 1.08 | 1.11 |
| *NADH-GOGAT2* | 1.23* | 0.28** |  | 2.47** | 0.50** |  | 3.01** | 1.12 |  | 0.43** | 0.67** |
| *Rubisco* | 0.33** | 0.23** |  | 0.68* | 1.12 |  | 1.19* | 0.22** |  | 0.88 | 0.82* |
| *Ppc1* | 0.33** | 0.33** |  | 0.70* | 0.99 |  | 2.91** | 2.25* |  | 1.14 | 1.99** |
| *Ppc2* | 0.54** | 0.24** |  | 1.03 | 0.60** |  | 6.27** | 1.72 |  | 1.26 | 2.39** |
| *Ppc3* | 0.36** | 0.25** |  | 1.03 | 1.30 |  | 0.30** | 0.44** |  | 0.15** | 0.29** |
| *Ppc4* | 0.60** | 0.22** |  | 1.04 | 0.89* |  | 1.27* | 0.33** |  | 0.87 | 0.88 |
| *Ppc6* | 0.95 | 0.34** |  | 0.91 | 1.44* |  | 1.80** | 0.56** |  | 0.61** | 0.82* |
| *Ppc7* | 0.67** | 0.22** |  | 1.06 | 1.11 |  | 3.32** | 1.19* |  | 0.67** | 1.03 |

|  | 1×N | | | | |  | 5×N | | | | |
| --- | --- | --- | --- | --- | --- | --- | --- | --- | --- | --- | --- |
|  | Root | |  | Leaf | |  | Root | |  | Leaf | |
|  | OX-*GS1;1* | OX-*GS1;2* |  | OX-*GS1;1* | OX-*GS1;2* |  | OX-*GS1;1* | OX-*GS1;2* |  | OX-*GS1;1* | OX-*GS1;2* |
| *NRT1;1* | 2.79** | 1.31* |  | 1.51** | 1.15 |  | 2.46** | 0.77 |  | 4.21** | 1.56** |
| *NRT1;2* | 2.06** | 1.50* |  | 1.09 | 0.35** |  | 1.55** | 0.52** |  | 0.59* | 0.33** |
| *NRT2* | 1.65** | 1.32 |  | 3.15** | 1.62 |  | 0.80* | 0.10** |  | 0.83 | 0.60 |
| *NR1* | 1.23 | 1.03 |  | 0.74 | 5.43** |  | 1.91** | 1.47** |  | 0.76** | 1.51** |
| *NR2* | 3.25** | 1.29** |  | 1.02 | 1.21 |  | 1.04 | 0.73 |  | 1.38** | 0.84* |
| *GS1;1* | 23.58** | 0.62* |  | 9.38** | 0.22** |  | 2.23** | 0.57** |  | 1.62* | 0.63* |
| *GS1;2* | 1.64** | 2.05** |  | 1.38 | 24.82** |  | 1.55* | 4.29** |  | 2.45** | 38.17** |
| *GS1;3* | 1.76** | 1.67** |  | 0.92 | 1.15 |  | 2.06** | 0.46** |  | 2.39** | 0.42** |
| *GS2* | 1.92** | 0.89 |  | 1.19 | 0.64** |  | 0.70* | 0.46** |  | 2.64** | 1.13* |
| *Fd-GOGAT1* | 2.40** | 1.18 |  | 1.00 | 0.71** |  | 1.79* | 1.01 |  | 1.78* | 0.85 |
| *Fd-GOGAT2* | 2.08** | 1.18 |  | 1.21 | 0.88 |  | 1.38** | 0.41** |  | 1.37* | 0.74* |
| *NADH-GOGAT1* | 2.23** | 0.98 |  | 1.02 | 1.14* |  | 1.13 | 0.60* |  | 1.13 | 0.78 |
| *NADH-GOGAT2* | 0.68** | 0.35** |  | 1.70** | 1.84** |  | 0.18** | 0.08** |  | 2.84** | 0.60* |
| *Rubisco* | 3.20** | 1.22 |  | 1.24* | 0.66** |  | 1.81** | 0.79** |  | 2.23** | 1.31* |
| *Ppc1* | 0.28** | 0.22** |  | 1.07 | 1.08 |  | 1.11 | 0.81 |  | 2.23** | 1.56* |
| *Ppc2* | 1.32 | 1.06 |  | 1.29* | 1.02 |  | 0.74** | 0.39** |  | 2.13** | 1.54** |
| *Ppc3* | 2.20** | 1.57* |  | 1.74** | 1.37* |  | 3.11** | 0.86 |  | 5.85** | 1.19 |
| *Ppc4* | 1.35* | 0.89 |  | 1.89** | 1.20* |  | 2.13** | 0.95 |  | 2.89** | 1.17 |
| *Ppc6* | 3.39** | 1.59* |  | 1.07 | 0.74* |  | 2.03** | 1.15 |  | 3.06** | 1.46** |
| *Ppc7* | 0.71* | 0.76* |  | 0.90 | 1.55* |  | 1.53** | 0.66** |  | 2.41** | 0.77* |

Values are mean from three independent experiments using three randomly mixed plant materials. *, ** indicate the significant differences at the level of P=0.05 and P=0.01, respectively. NRT: nitrate transporter; NR: nitrate reductase; GS: glutamine synthetase; GOGAT: glutamate synthase; RUBISCO: Ribulose-1,5-bisphosphate carboxylase/oxygenase; PEPC: phosphoenolpyruvate carboxylase.
